# Optimization of Cinnamon Planting Density in Coconut Intercropping System in India

**DOI:** 10.1155/sci5/6485499

**Published:** 2025-07-20

**Authors:** Subramanian Periasamy, Surekha, Ravi Bhat, Anok Uchoi

**Affiliations:** ^1^Division of Crop Production, ICAR-Central Plantation Crops Research Institute, Kasaragod 671124, Kerala, India; ^2^Division of Crop Production, ICAR-CPCRI RC Kahikuchi, Guwahati, Assam 781017, India

**Keywords:** cinnamon, coconut, India, planting density, planting method, system productivity

## Abstract

The study aimed to explore the potential of cinnamon intercropping as a vertical expansion strategy with increased planting density to increase cinnamon production and address the surge in global demand for this valuable spice crop. Despite lower individual plant quill yield, the high-density pentagonal method at 0.6 m × 1.2 m spacing with 7290 plant population h^−1^a resulted in high yield of 632 and 979 kg of cinnamon per hectare during first and second harvests, respectively, outperforming the control spacing (2.0 m × 2.0 m, 1404 population ha^−1^). Furthermore, the coconut + cinnamon intercropping system with spacing of 0.6 m × 1.2 m resulted in significantly higher system productivity of coconut equivalent yield of 55,766 nuts per hectare per year (average of two years) compared to other spacing treatments, showcasing its potential for enhanced cultivation and productivity. Therefore, the study concluded that cinnamon is a promising and economically feasible intercrop in coconut plantations when planted using the high-density pentagonal planting method with a closer spacing of 0.6 m × 1.2 m with 7290 plant population ha^−1^.

## 1. Introduction

Spices refer to dry aromatic parts of plants, which include seeds, fruits, roots, and leaves and are known to be high-value and low-volume commercial products in the world market [1]. Globally, the food industry is the fastest growing industry, relying mainly on spices as producers of taste and flavor, and they also improve the nutritional quality of the diet as they are the source of natural colors and flavors [2]. They also play an important role in the prevention and treatment of many diseases. Due to its neutraceutical [3], microbial, and antioxidant properties [4], the ability of spices to promote digestion and weight loss has given them a special place in pharmaceuticals [5]. Cinnamon (*Cinnamomum verum* J. Pres) is one of the important spice crops. Its habitat is a tropical forest, and although the height is about 10 m, it grows as a bush under less than 3 m high. The dried inner bark, bark oil, bark oleoresin, and leaf oil are the economically important products of cinnamon.

Cinnamon has been reported to contain cinnamaldehyde, eugenol, cinnamic acid, cinnamyl alcohol, and coumarin. More than 80 compounds were identified from different parts of cinnamon [6, 7]. It has been widely utilized in food, beverage, pharmaceutical, and cosmetics industries [8] since the dawn of humanity. Increased use of this crop in the pharmaceutical and cosmetics industries sectors has created a surge in demand for it globally in recent years [9, 10]. According to the Food and Agriculture Organization of the United Nations, China was the major producer of cinnamon (96,554 t), followed by Indonesia (56,664 t), Vietnam (45,680.3 t), Sri Lanka (23,729.8 t), and Madagascar (3778.7 t). In India, cinnamon is mainly cultivated in Cannanore (Kerala), South Kanara (Karnataka), Nilgiris, Lower Palani, Courtallam, and Kanyakumari (Tamil Nadu). Although cinnamon is an older known spice crop in India, its cultivation is limited to very little area with deficient production. According to the Directorate of Arecanut and Spices Development (DASD) report 2021, India barely produces 72 t of cinnamon from 202 ha cultivated area; however, the consumption by the large population necessitates import of 2635 t of cinnamon to satisfy the domestic demand.

Several species belonging to the genus *Cinnamomum* are traded as cinnamon in the local and international markets in different parts of the world [11, 12]. Of them, *Cinnamomum verum* is considered superior to other species due to its unique biochemical properties.

Cinnamon is sold in the market that is adulterated with cassia cinnamon (*Cinnamomum cassia)* bark, having high coumarin content (2.88–4.82 mg g^−1^) [13], which is above the permissible limit (2 mg kg^−1^). European health agencies have recently issued a warning against consuming high amounts of coumarin because although coumarin has a positive impact at lower concentrations [14, 15], it could be a carcinogen and toxic at higher concentrations [16]. As a result, there is enormous potential to expand true cinnamon (*Cinnamomum verum)* cultivation in traditional and nontraditional areas of the cinnamon-growing countries. However, monocropping of cinnamon is not feasible and advisable as agricultural land has diminished over the years. Vertical expansion strategy must be employed and encouraged over horizontal expansion and intercropping system is one such potential vertical expansion strategy. Perennial plantation gardens such as coconut, arecanut, rubber, oil palm, and cashew can be successfully exploited for growing cinnamon as intercrop.

Coconut (*Cocos nucifera* L.) is one of the most significant plantation crops, which is well known as “*Kalpavriksha*,” “*Tree of Heaven*,” and “*Tree of life*” because of its innumerable benefits to the mankind. It is mostly grown in tropical and subtropical climates worldwide. At global level, area under coconut cultivation is 12.26 m ha, with Indonesia (3.40 m ha), Philippines (3.65 m ha), India (2.17 m ha), Sri Lanka (0.44 m ha), and Thailand (0.12 m ha) making up 75% of the total area. The wider spacing of coconut (7.5 m × 7.5 m) and fibrous rooting pattern in which 75% of the active roots are mainly confined to 1.8 m width and 1.0 m depth will leave the land unutilized, and hence, about 60% of the coconut garden is available for growing other intercrops. Similarly, the canopy structure and phyllotaxy of the palm allow solar radiation to penetrate to the ground (50%–60% light availability) [17, 18]. Thus, a number of coconut-based cropping system models involving annuals, biennials, perennials, and combinations of both annuals and perennials have been developed across the coconut-growing regions globally to name a few such as forages in Western Samoa [19]; cocoa, cashew, black pepper in Brazil [20]; vegetables [21, 22], tubers [23, 24] pulses [25], flowers [26] in India; citrus in Ghana [27]; and multiple crops in Philippines [28] and Sri Lanka [29]. The tap root system of cinnamon enables it to withstand moisture stress and extract moisture from deeper layers of the soil. Thus, cinnamon can be termed as climate-resilient crop. The ability of cinnamon to grow and yield satisfactorily under moderate shade has made it possible to grow it as a perennial intercrop under plantations like rubber (*Hevea brasiliensis* Muell. Arg.) [30]. This character of cinnamon along with its coppicing ability has opened up new avenues for expanding the area under this crop. For northeastern region of India, cinnamon air layers of Nithyasree and Navasree varieties were developed in ICAR-CPCRI RC Kahikuchi and supplied to the farmers.

The current cultivation practices result in less yield with low- to medium-grade quills. Thus, there is a need to upgrade or modify the current cultivation practices to enhance the production of long, straight stems with medium girth for obtaining higher-grade quills. Accordingly, the present study was undertaken with the objective to study the effect of high-density pentagon planting methods with different spacing as intercrop in the coconut garden on the yield and quality of cinnamon quills.

## 2. Materials and Methods

### 2.1. Experimental Site

The field experiment was carried out for three consecutive years during 2019‐20 and 2021‐2022 at the Research Farm, ICAR-Central Plantation Crops Research Institute, Kasaragod, Kerala, India. The experimental site is situated at 12° 30′ N latitude and 75° 00′ E longitude with an altitude of 10.7 m above mean sea level. The climate of the site is typical warm humid tropical. The mean rainfall, relative humidity, and maximum and minimum temperature during the growing seasons were 4499.5 mm, 70.1%, and 31.8°C and 22.8°C, respectively. The soil is red sandy loam soil (pH: 5.07). At the commencement of the experiments, the soil was characterized as having organic C (0.43%), low available N (223.63 kg ha^−1^), high available P (151.47 kg ha^−1^), and available K (191.37 kg ha^−1^). The bulk density, particle density, water-holding capacity, porosity, and cation exchange capacity (CEC) were 1.35 g cm^−3^, 2.36 g cm^−3^, 27.56%, 34.12%, and 5.89 cmol kg^−1^, respectively.

### 2.2. Treatment Details

**Figure 1:**
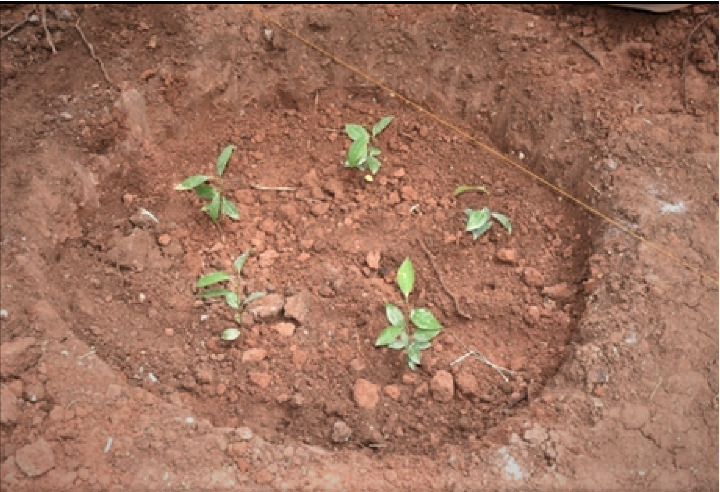
Using the pentagonal model, five seedlings were planted in each pit.

The experiment was conducted in randomized block design with six spacing treatments, namely 1.8 m × 1.2 m, 1.5 m × 1.2 m, 1.2 m × 1.2 m, 0.9 m × 1.2 m, 0.6 m × 1.2 m, and control −2.0 m × 2.0 m (single plant per pit) with six replications, and high-density pentagonal method (Figure 1) was taken up for first five treatments (1.8 m × 1.2 m, 1.5 m × 1.2 m, 1.2 m × 1.2 m, −0.9 m × 1.2 m, 0.6 m × 1.2 m), and control (2.0 × 2.0 m, single plant per pit) is the recommended traditional method of planting with one plant per pit.

### 2.3. Cultivation, Harvest, and Data Collection

The interspace was ploughed to a fine tilth in order to take up cinnamon planting in a 52-year-old coconut plantation (variety West Coast Tall) planted at a spacing of 8 m × 8 m. To intercrop cinnamon within coconut plantations, 60% of the interspace area in the coconut garden was utilized for planting. As per the spacing treatment, the area for planting was marked and circular pits of 0.5 m radius with 0.5 m depth were opened. Excavated top soil was added to the pits to a depth of 0.25 m, and dried cow dung (1 kg plant^−1^) + neem cake (250 g plant^−1^) + rock phosphate (100 g plant^−1^) were mixed thoroughly into the soil. Fabricated pentagon model was placed inside the pit, and planting of 1-year-old cinnamon seedlings (variety Navasree) was taken up (Figure 1). The basin was mulched with dried leaves, and protective irrigation (from January to May 10 L per pit at 15-day interval) was given during summer season. The fertilizer was applied @ 20:18: 25 g N, P_2_O_5_, and K_2_O per plant for the first year; 40: 36: 50 g N, P_2_O_5_, and K_2_O during the second year; and 60:54: 75 N, P_2_O_5_, and K_2_O per plant for the third year. Fertilizer was applied in two equal splits: one during the onset of monsoon (June) and another during the first fortnight of September. During September, digging of the soil around the bushes was taken up and chemical fertilizer was applied and 1 kg plant^−1^ of organic manure was applied over that and incorporated into the soil. Two weeding systems in a year during July–August and October–November were carried out.

The coconut palms were irrigated with drip irrigation system at 66% pan evaporation (32 L palm^−1^ day^−1^) from November to May, and the fertilizers were applied based on soil test value. The N, P, and K were applied in the form of urea, rock phosphate, and muriate of potash, respectively, in two splits. The first dose, that is, one-third (33%) of the recommended dose of fertilizer was applied during the beginning of southwest monsoon (May–June), and the remaining two-third (66%) of the recommended dose of fertilizer was applied during receding monsoon (September–October). Fertilizers were applied by being broadcast in circular basins of 1.8 m around the palm.

**Figure 2:**
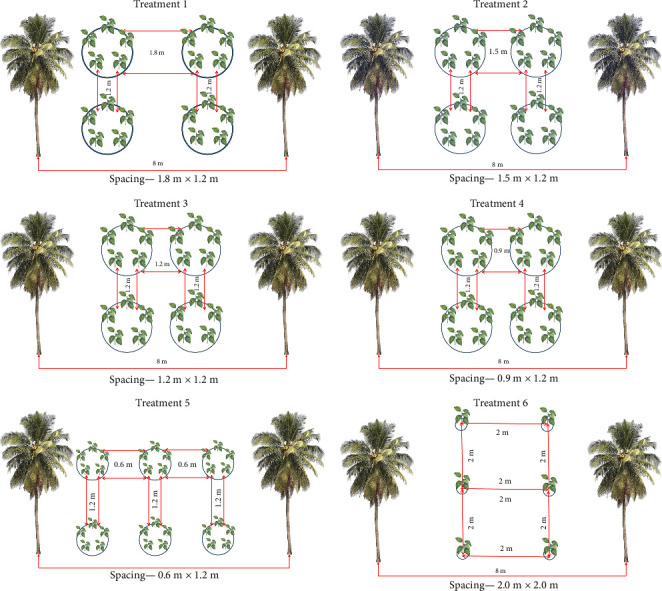
Schematic representation of the coconut + cinnamon intercropping system in different spacing.

The planting density of cinnamon per ha was 5280, 5280, 5280, 5280, 7290, and 1404 plants at 1.8 m × 1.2 m, 1.5 m × 1.2 m, 1.2 m × 1.2 m, 0.9 m × 1.2 m, 0.6 m × 1.2 m, and 2.0 m × 2.0 m spacing, respectively. For the first four spacing treatments, the plant density is uniform because the spacing between two pits within a row is fixed, that is, 1.2 m, and between two rows the spacing is changed, that is, 0.9 m–1.8 m; with this, there is no increase in cinnamon row numbers. However, with 0.6-m spacing between the two rows of cinnamon, one more row of cinnamon was accommodated. It hence resulted in 3 rows of cinnamon between two rows of coconut palms, and the population number was increased to 7290. The schematic representation of different plant spacing was illustrated in Figure 2.

The observations on growth attributes of cinnamon like plant height stem girth (10 cm above the ground level) and number of branches were recorded during May 2020, 2021, and 2022. After the plants reached 2 years of age, the first harvest was taken up by coppicing during June–July to a height of about 10 cm from the ground, and the stem attained 10 cm and above thickness with uniform brown color, which is considered an ideal stage for bark extraction. The second harvest was taken up during the subsequent year. After first harvest, two shoots per plants were retained, and for the second harvest, about three plants per pit were ready to harvest. Thus, the second year yield includes three plants per pit and two shoots per plant. After coppicing/harvesting, the stump was covered by earthing up. The side branches along with leaves were removed from the harvested stem. The stems were scraped with the help of knife/peeler to remove the brown surface. The scrapped portion was rubbed gently with a brass rod to facilitate easy peeling by removing the moisture held between pith and bark and moisture in the bark. A longitudinal slit was made, and the bark was removed by working the knife between the bark and the pith. The shoots harvested during morning are peeled on the same day. The peels were dried first in shade for a day and then in sunlight for 4 days. During drying, the bark contracts and assumes the shape of a quill by rolling. The smaller quills are inserted into larger ones to form compound quills, and the dry weight is recorded and considered as yield.

The yield data of coconut were recorded at each harvest from the palms, and annual yield per palm was computed. The system productivity was calculated from the coconut equivalent yield of cinnamon with the formula:

(1) $$\begin{array}{c} \text{System productivity}=\text{Coconut yield}+\left(\frac{\text{Yield of cinnamon}\times \text{price of cinnamon}}{\text{price of coconut}}\right). \end{array}$$

### 2.4. Quality Analysis

The cinnamon quality parameters such as essential oil (%) and oleoresin (%) content were analyzed by following the standard procedure mentioned in American Spice Trade Association official analytical methods [31].

#### 2.4.1. Estimation of Essential Oil

A modified Clevenger device was used to estimate the amount of essential present in the cinnamon. A 25-g of cinnamon sample that had been powdered was placed in a 1-L round bottom flask, filled with water, filtered through a Clevenger trap and condenser, and then heated for 3 hours until boiling. The essential oil present in the sample comes out during boiling and being lighter in weight and immiscible with water, which forms a separate layer on top. This separated oil was collected in a centrifuge tube containing anhydrous sodium sulfate. The oil was kept in refrigerator while the analysis was being done. The percentage oil in the sample was calculated as follows:

(2) $$\begin{array}{c} \text{Essential oil }\left(\%\right)=\frac{\text{Volume of oil extracted in mL}}{\text{weight of sample in }g}*100. \end{array}$$

#### 2.4.2. Estimation of Oleoresin

A 10-g of finely ground cinnamon sample was taken in a column with cotton at the bottom. To this, 50 mL of acetone was added and the sample in the column was kept undisturbed overnight. The extract was drained into a preweighted beaker. This procedure was continued till the cotton became colorless. The solvent was dried in a water bath by evaporation. The amount of oleoresin present in the sample was determined by the difference in the weight of the beaker. The oleoresin content was calculated as

(3) $$\begin{array}{c} \text{Oleoresin }\left(\%\right)=\frac{\text{Weight of the residue in }g}{\text{Weight of the sample in }g}*100. \end{array}$$

### 2.5. Economic Analysis

The cost of cultivation, gross return, net return, and B : C (benefit : cost ratio) were computed treatment-wise for cinnamon, coconut, and cinnamon + coconut and are expressed in per hectare of coconut garden. The prices of the inputs that were prevailing at the time of their use and selling price for cinnamon quill and coconut in the market were taken into account, and economic analysis was made and is expressed in USD.

### 2.6. Statistical Analysis

The collected data from the experiments were statistically analyzed using analysis of variance (ANOVA) following the procedure described by Gomez and Gomez [32]. The level of significance used in “*F*” and “*t*” tests was *p* = 0.05. Critical difference values were calculated wherever the “*F*” test was significant. The statistical analysis was performed using standard statistical package using SPSS.

## 3. Result

### 3.1. Growth Parameters

The effect of different spacings on growth parameters is presented in Table 1, Tables 2, and 3, suggesting spacing had significant influence on growth parameters. Among the different treatments tried, 0.6 m × 1.2 m spacing recorded the most height (172.9 and 429.4 cm, respectively) and significantly differed from the spacing, such as 1.8 m × 1.2 m (152.9 and 360.5 cm, respectively), and 2 m × 2 m (139.8 and 401.2 cm, respectively), but was on par with 0.9 m × 1.2 m (169.7 and 413.5 cm, respectively) and 1.2 m × 1.2 m (165.6 and 408.7 cm, respectively) during first and second years, respectively. During the third year, there was no significant difference among the treatments, and the plant height ranged from 311.1 to 360.1 cm (Table 1). Similarly significantly higher number of branches recorded under treatment 0.6 m × 1.2 m (13.3 and 22.2, respectively) over 1.8 m × 1.2 m (10.8 and 18.7, respectively) and 2.0 m × 2.0 m (10.2 and 18.5, respectively) during first and second years, respectively. During the third year, there is no significant difference among the treatments, and the number of branches ranged from 16.6 to 22.1 (Table 2). However, higher girth was recorded under wider spacing, that is, 2 m × 2 m (16.4 cm) over other treatments followed by 1.8 m × 1.2 m (15.0 cm), 1.5 m × 1.2 m (14.8 cm), and 1.2 m × 1.2 m (14.3 cm) during second year and was on par with each other during first and third years (Table 3). Significantly lower stem girth was recorded in treatment 0.6 m × 1.2 m (13.3 cm) and was on par with 0.9 m × 1.2 m (13.5 cm) during the second year.

### 3.2. Yield and Quality Parameters

Cinnamon yield and yield parameters are presented in Table 4. Different spacing treatments had a significant influence on the length of matured stem for extraction of bark, quill yield plant^−1^ and ha^−1^. Higher length of matured shoot for extraction of bark was recorded under 0.6 m × 1.2 m (3.87 m) compared to 1.8 m × 1.2 m (3.10 m) and 1.5 m × 1.2 m (3.24 m) and on par with 2 m × 2 m (3.68 m), 0.9 m × 1.2 m (3.58 m), and 1.2 m × 1.2 m (3.45 m). With regard to individual plant quill yield, an increase in individual plant quill yield, in widely spaced (2.0 × 2.0 m) trees, recorded significantly higher individual plant quill yield (229.3 and 263.8 g plant^−1^, respectively) over other treatments during first and second harvests, respectively. However, with regard to per-hectare quill yield, significantly higher quill yields of 631.9 and 979.0 kg ha^−1^, respectively, during first and second harvests (after the first harvest, per plant only two shoots were retained for subsequent harvests) were recorded in closely spaced high-dense plants (0.6 m × 1.2 m with 7290 population) over other treatments, and significantly lower per-hectare quill yields of 321.6 and 370.4 kg ha^−1^, respectively, were recorded in widely spaced (2.0 m × 2.0 m with 1404 population).

Effect of cinnamon intercropping on coconut yield and system productivity is presented in Table 5. There is no significant influence of cinnamon intercropping in coconut garden with different spacing, and the yield ranged from 116 to 123 nuts palm^−1^ year^−1^, 122 to 137 nuts ha^−1^ year^−1^, and 114 to 130 nuts ha^−1^ year^−1^ during 2019-20, 2020-21, and 2021-22, respectively. If we consider the coconut + cinnamon system productivity, the spacing 0.6 m × 1.2 m recorded significantly higher system productivity of 55,766 nuts ha^−1^ over other treatments and significantly lower system productivity recorded by treatment 2.0 × 2.0 m (37,170 nuts ha^−1^), which was on par with 1.8 m × 1.2 m (39,836 nuts ha^−1^) and 1.5 m × 1.2 m (40,567 nuts ha^−1^).

### 3.3. Cinnamon Quality

**Figure 3:**
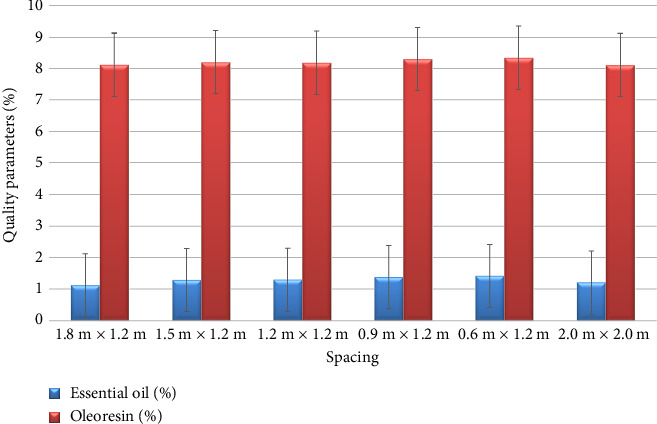
Effect of spacing on the quality parameters of cinnamon bark intercropped in coconut.

The cinnamon quality parameters, such as essential oil (%) and oleoresin (%) as influenced by spacing treatments are depicted in Figure 3. The results on quality parameters revealed that there was no significant influence of plant spacing on the essential oil and oleoresin contents. However, the essential oil content varied considerably from 1.12% to 1.42% and the oleoresin content from 8.11% to 8.34%.

### 3.4. Economics

**Figure 4:**
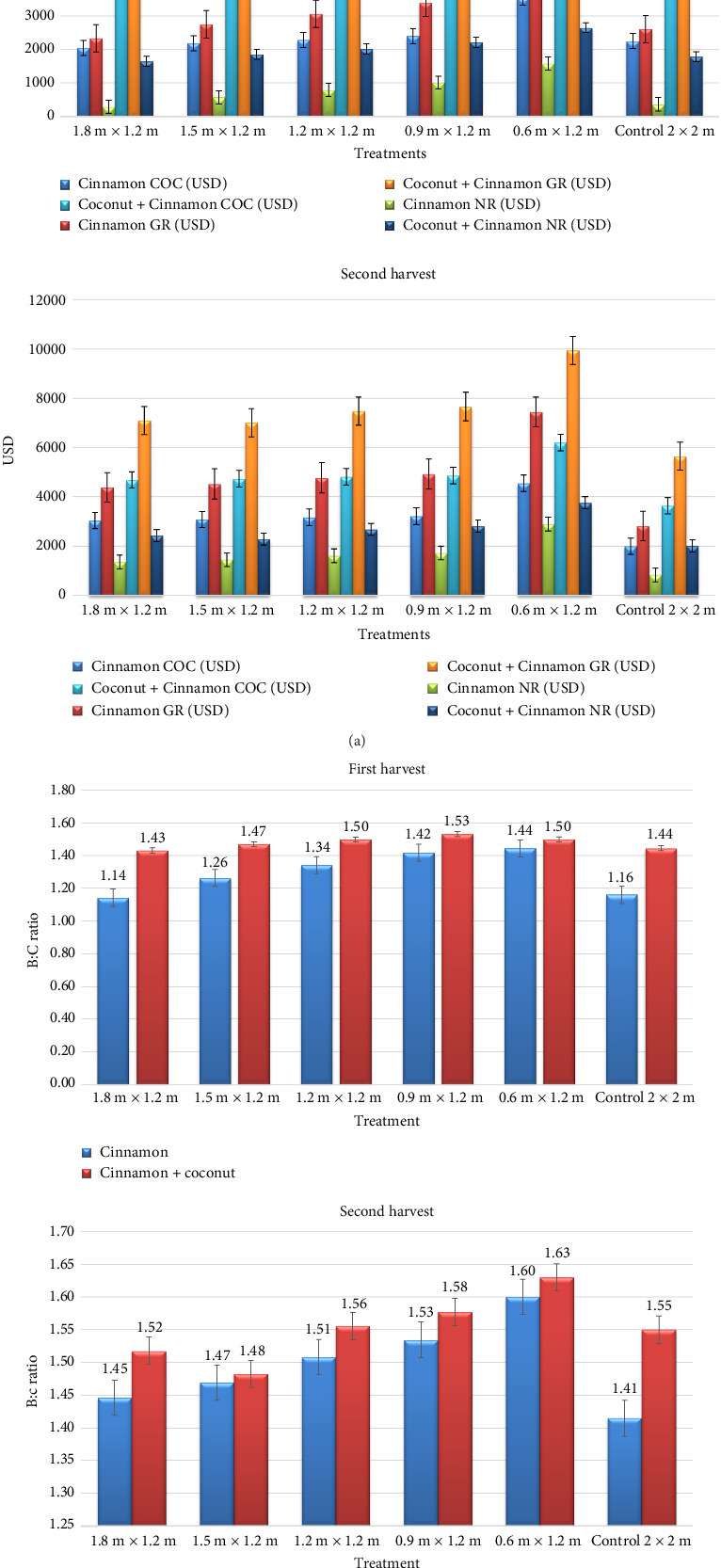
(a) Effect of spacing on the cost of cultivation, gross returns, and net returns of cinnamon and coconut + cinnamon cropping system (USD ha^−1^) during first and second harvests. (b) Effect of spacing on the benefit: cost ratio of cinnamon and coconut + cinnamon cropping system during first and second harvests.

The economic analysis of cinnamon and coconut + cinnamon intercropping system is presented in Figure 4. With regard to cinnamon economic analysis, higher cost of cultivation (3550 and 4552 USD, respectively) was incurred in the treatment 0.6 m × 1.2 m during first and second harvests followed by other high-density pentagon planting method, that is,0.9 m × 1.2 m (2401 and 3210 USD, respectively); 1.2 m × 1.2 m (2288 and 3160 USD, respectively); 1.5 m × 1.2 m (2185 and 3079 USD, respectively); and 1.8 m × 1.2 m (2044 and 3028 USD). Lower cost of cultivation was recorded in control, that is, 2.0 m × 2.0 m (2249 and 1990 USD, respectively). Higher gross return (5128 and 7441 USD, respectively) and net return (1578 and 2889 USD, respectively) were obtained when the cinnamon was planted with 0.6 m × 1.2 m spacing in high-density pentagon method during first and second harvests followed by 0.9 m × 1.2 m (3403 and 4925 gross return; 1002 and 1715 USD net returns, respectively). Lower gross return (2335 and 4378 USD, respectively) and net return (291 and 1350 USD, respectively) were obtained when the cinnamon was planted at 1.8 m × 1.2 m spacing with high-density pentagon method. The economics of the whole system is an important parameter for the success of any production technology. Even though the higher cost of cultivation of the system was recorded in 0.6 m × 1.2 m spacing in high-density pentagon method treatment (5310 and 6200 USD, respectively), it also recorded higher gross return (7959 and 9947 USD, respectively) and net return (2649 and 3746 USD, respectively). Lower cost of cultivation (3639 USD), gross return (5640 USD), and net return (2002 USD) were recorded in control −2.0 m × 2.0 m, single plant per pit during second harvest; however, during first harvest, lower cost of cultivation (3804 USD), gross return (5445 USD), and net return (1641 USD) recorded in 1.8 m × 1.2 m spacing with high-density pentagon method of planting.

## 4. Discussion

### 4.1. Growth Parameters

The results on growth parameters of cinnamon suggest that closer spacing leads to increased height and increased number of branches but decreased in girth. Planting density will have a large effect on any plant's morphology and its stem bark content. Phillips [33] reported that the closest-spaced citrus trees in Florida, 702 ha^−1^, were significantly tall compared to 16 trees ha^−1^. Similarly, Ranatunga et al. [34] reported that closer spacing recorded increased cinnamon plant height as they seek sunlight. In highly dense system, the cinnamon shoot height tends to increase because of the competition caused by neighboring plant's mutual shading, which occurred due to the reduction of the red: far-red ratio within canopies and etiolating effect [35–37]. Dao et al. [38] reported that closer spacing encourages formation of tall, straight trunks with as few branches as possible. Usman [39] reported that wider spacing recorded higher girth. Kumar and Singh [40] reported that the girth and volume of trees showed a decreasing trend with increasing tree density. In contrast, tree height increased with increasing tree density in Allahabad Safeda guava.

### 4.2. Yield and Yield Parameters

The results on quill yield indicated that high-density population with closer spacing led to an increased quill yield ranging from 5.6% to 96.5% in the first harvest and from 55.5% to 83.3% in second harvest compared to the control. The improved yield and yield parameters of cinnamon in high-density population with closer spacing is because of the fact that the optimum spacing enables maximum interception of solar radiation, which in turn ensures more efficient photosynthetic activities resulting in higher availability of net photosynthesis. Similar findings were reported by Singh [41], in Guava. Fang et al. [42] reported 67.5% increased bark yield of *Pteroceltis tatarinowii* recorded when the planting density was increased from 2500 stumps ha^−1^ to 4200 stumps ha^−1^. Though the control treatment 2 m × 2 m recorded higher quill yield per plant, total estimated yield recorded higher under closer spacing. Closer spacing led to increased length of mature shoot and helped in extracting more bark yield under 0.6 m × 1.2 m spacing (631.92 and 979.0 kg ha^−1^, respectively) treatment, which was significantly higher over other treatments during both first and second harvests. Population was higher (7290 plants ha^−1^ of coconut garden) under 0.6 m × 1.2 m spacing treatment and that significantly contributed to the higher yield from other treatments (1404–5280 plants ha^−1^ of coconut garden). Similarly, Cockerham [43] in California reported that the tree size such as the height, diameter, volume, and weight of individual tree of River red gum, *Eucalyptus camaldulensis,* was greater in the wide spacing of the lower planting density, while per-acre volume and weight yields were greater at the higher planting density. Tanasombat et al. [44] also reported that in Thailand, the gross production of paper mulberry (*Broussonetia papyrifera* and *B. kazinoki*) wood by stands planted at close spacing is greater than that of those planted at wide spacing.

From the whole system productivity, that is, coconut + cinnamon, it was clear that cinnamon intercropping did not exhibit any competitive effect on coconut; however, the yield started increasing over pre-experimental period as the cinnamon plants with tap root systems and coconut palms with fibrous roots with rooting patterns at different soil layers do not display competition. The density of plants in agroforestry systems leads to competition for water and nutrients, especially when the roots of the main and intercrop occupy same soil layer [45].

Even though the actual impact of any agromanagement practice would normally start from the fourth year onwards [46], a significant influence on the overall system productivity was observed. Cinnamon pentagonal planting method with a spacing of 0.6 m × 1.2 m recorded significantly higher system productivity of 55,766 nuts ha^−1^ year^−1^ over other spacing options. Significantly lower system productivity of 37,170 nuts ha^−1^ year^−1^, 39,836 nuts ha^−1^ year^−1^, and 39,836 nuts ha^−1^ year^−1^ was recorded with wider spacing of 2.0 × 2.0 m, 1.8 m × 1.2 m, and 1.5 m × 1.2 m, respectively. This may be due to the higher efficiency of utilization of basic resources of crop production such as land, solar radiation, and water [17] as observed in other studies [47]. Red / far-red light ratio of light available under rubber tree canopy was low at high-density planting [35]. Bark weight [48] and plant architecture [49] of cinnamon can be manipulated by spatial pattern of planting, choice of planting material, and harvest intervals. Hemp plants grown at higher densities among the treatments 30, 90, and 270 plants per square meter resulted in self-thinning leading to reduction of bark content [50]. Lowest row spacing attempted at 35.5 cm recorded the highest yield of bark per hectare in kenaf [51]. This clearly indicated that growing of cinnamon as intercrop even under higher density has no negative effect on the coconut yield. The results on essential oil (%) and oleoresin (%) obtained in the present study are in accordance with the previous report on cinnamon quality [52].

### 4.3. Economics

Higher cost of cultivation in closer spacing of 0.6 m × 1.2 m with high-density pentagon method of planting was mainly due to higher number of population accommodated compared to other treatments and, similarly, lower cost of cultivation recorded in control (2.0 m × 2.0 m with single plant per pit) because of the lower number of population accommodated in the treatment. Here, the number of plant populations is only the variable affecting the cost of cultivation as all other management practices followed were the same across the treatments including control. Higher gross and net returns recorded in closer spacing of 0.6 m × 1.2 m with high-density pentagon method of planting were mainly due to the cumulative effect of individual bark yield and increased plant population. As there was no treatment imposition on the coconut, there is no difference in cost of cultivation across the treatments studied, and also there is no much difference in gross and net returns. Compared to monocrop of coconut, the system of whole coconut + cinnamon is more economical. Coconut + cinnamon system as a whole recorded 25.1%–114.4% higher net return over coconut cultivation as monocrop. In the coconut + cinnamon system, cinnamon planting with a closer spacing of 0.6 m × 1.2 m with high-density pentagon method of planting recorded 32.8% and 46.6% increases in net return during first and second harvests, respectively, over control, followed by 0.9 m × 1.2 m with high-density pentagon method of planting (38.1% and 35.4%, respectively). Similarly, Pathirathna et al. [53] reported that the net revenue in the rubber plantation with 496 trees ha^−1^ population density without cinnamon recorded a deficit of Rs. 36947 ha^−1^ compared to a gain of 248,000 ha^−1^ in the same treatment with cinnamon as intercrop.

Shindae et al. [54] reported that net economic returns indicated that nutmeg, cinnamon, and clove could be grown successfully as intercrops in well-spaced coconut gardens in the Konkan region of Maharashtra state. Nagawekar et al. [55] reported that nutmeg intercropping recorded 177% higher net returns than monocropping of coconut followed by cinnamon (103%), clove (45%), and black pepper (37%), The present study of cinnamon intercropping in coconut also confirmed that cinnamon is a promising intercrop in the coconut garden. The economics of high-density rubber-based agroforestry systems are higher than low-density systems [56].

## 5. Conclusion

This present preliminary study conducted at ICAR-CPCRI Kasaragod, Kerala, India, revealed that cinnamon is a promising intercrop in coconut gardens with high-density pentagonal method of planting with high population of 7290 per hectare of coconut garden for increased cinnamon quill yield (631.9 and 979.0 kg ha^−1^, respectively, during first and second harvests) and is also economically feasible with increased B:C ratio (1.5 and 1.6, respectively, during first and second harvests) over normal spacing of 2 m × 2 m. This will not only help in increasing the overall system productivity in coconut garden, but also helps in vertical expansion of area under cinnamon cultivation. However, performance of cinnamon for long period needs to be studied. Further, there is a need to work out the package of practice including nutrient and water management for cinnamon under high-density planting condition.

## Figures and Tables

**Table 1:** Effect of spacing on the cinnamon plant height (cm) in coconut garden.

Spacing of cinnamon (m)	Plant height (cm)		
	1st year (2019-20)	2nd year (2020-21)	3rd year (2021-22)
1.8 × 1.2	152.9^b^	360.5^c^	311.1
1.5 × 1.2	161.6^ab^	378.1^bc^	342.3
1.2 × 1.2	165.6^a^	408.7^ab^	350.4
0.9 × 1.2	169.7^a^	413.5^ab^	324.6
0.6 × 1.2	172.9^a^	429.4^a^	360.1
2.0 × 2.0 (Control)	139.8^c^	401.2^b^	300.0
S. Em ±	3.9	8.2	12.9
CD (*p* = 0.05)	11.59	24.37	NS

*Note:* The means followed by the same lower letter do not differ significantly at the 0.05% probability.

**Table 2:** Effect of spacing on the cinnamon plant number of branches (no.) in coconut garden.

Spacing of cinnamon (m)	Number of branches		
	1st year (2019-20)	2nd year (2020-21)	3rd year (2021-22)
1.8 × 1.2	10.8^c^	18.7^b^	18.3
1.5 × 1.2	12.3^b^	20.8^a^	17.8
1.2 × 1.2	13.0^ab^	21.0^a^	16.6
0.9 × 1.2	13.1^ab^	22.0^a^	21.5
0.6 × 1.2	13.3^a^	22.2^a^	22.1
2.0 × 2.0 (Control)	10.2^c^	18.5^b^	17.5
S. Em±	0.30	0.60	2.07
CD (*p* = 0.05)	0.89	1.78	NS

*Note:* The means followed by the same lower letter do not differ significantly at the 0.05% probability.

**Table 3:** Effect of spacing on the cinnamon plant girth (cm) in coconut garden.

Spacing of cinnamon (m)	Girth (cm)		
	1st year (2019-20)	2nd year (2020-21)	3rd year (2021-22)
1.8 × 1.2	7.20	15.0^b^	23.1
1.5 × 1.2	7.00	14.8^b^	23.8
1.2 × 1.2	6.80	14.3^bc^	22.6
0.9 × 1.2	6.60	13.5^c^	20.6
0.6 × 1.2	6.50	13.3^c^	22.3
2.0 × 2.0 (Control)	8.30	16.4^a^	22.5
S. Em ±	0.36	0.31	2.03
CD (*p* = 0.05)	NS	0.93	NS

*Note:* The means followed by the same lower letter do not differ significantly at the 0.05% probability.

**Table 4:** Effect of spacing on the bark yield of cinnamon in coconut garden.

Spacing of cinnamon (m)	Length of matured shoot length for extraction of bark (m) (average of 2 harvests)	Quill yield			
		1st harvest		2nd harvest	
		Per plant (g)	Total yield (kg ha^−1^)	Per plant (g)	Total yield (kg ha^−1^)
1.8 × 1.2	3.10^b^	54.5^c^	287.76^c^	109.2^b^	576.0^b^
1.5 × 1.2	3.24^b^	64.3^c^	339.68^bc^	112.7^b^	595.0^b^
1.2 × 1.2	3.45^ab^	71.7^bc^	378.13^bc^	118.8^b^	627.0^b^
0.9 × 1.2	3.58^ab^	79.5^bc^	419.41^b^	122.7^b^	648.0^b^
0.6 × 1.2	3.87^a^	86.8^b^	631.92^a^	123.7^b^	979.0^a^
2.0 × 2.0 (Control)	3.68^ab^	229.3^a^	321.6^c^	263.8^a^	370.4^c^
S. Em±	0.18	7.02	27.43	12.12	35.7
CD (*p* = 0.05)	0.54	21.34	80.34	35.5	104.56

*Note:* (1) The means followed by the same lower letter do not differ significantly at the 0.05% probability. (2) Two shoots per plant were retained after the first harvest and during the second harvest, and three plants per pit were attained in the harvesting stage.

**Table 5:** Effect of cinnamon intercropping on coconut yield (nuts/palm/year) and system productivity.

Spacing of cinnamon (m)	Pre-experimental (average of two years) 2017-18 and 2018-19	Nut yield (no. of nuts palm^−1^ year^−1^)			System productivity (no. of nuts ha^−1^) (Average of 2020-21 and 2021-22)
		1st year (2019-20)	2nd year (2020-21)	3rd year (2021-22)	
1.8 × 1.2	102	120	134	125	39836^c^
1.5 × 1.2	99	119	131	114	40567^c^
1.2 × 1.2	96	118	129	125	42931^bc^
0.9 × 1.2	94	116	128	126	44495^b^
0.6 × 1.2	91	116	122	115	55766^a^
2.0 × 2.0 (Control)	109	123	137	130	37170^c^
S. Em±	4.4	2.50	3.28	2.72	1324.8
CD (*p* = 0.05)	NS	NS	NS	NS	3880.9

*Note:* The means followed by the same lower letter do not differ significantly at the 0.05% probability.

## Data Availability

All the data pertaining to manuscript are already presented in this manuscript. Furthermore, the data can be availed from the corresponding author on request basis.
